# Case report: Transformative potential of trastuzumab deruxtecan in the first line treatment for advanced HER2-mutated lung adenocarcinoma: a case report and clinical insights

**DOI:** 10.3389/fphar.2025.1564834

**Published:** 2025-04-25

**Authors:** Guanbin Huang, Tongtong Xia, Dongxiao Yang, Muwen Lin, Yiping Luo, Yuchan Li, Hualin Chen

**Affiliations:** Department of Lung Cancer, Affiliated Hospital of Guangdong Medical University, Zhanjiang, China

**Keywords:** HER2-mutant NSCLC, trastuzumab deruxtecan, antibody-drug conjugate, first line treatment, central nervous system metastases

## Abstract

The treatment of non-small cell lung cancer (NSCLC), particularly lung adenocarcinoma (LUAD), represents a significant oncological challenge due to its aggressive behavior, high metastatic potential and poor prognosis. HER2 mutation is a rare but critical oncogenic driver associated with resistance to traditional therapies among NSCLC subtypes, including chemotherapy and tyrosine kinase inhibitors (TKIs). Trastuzumab deruxtecan (T-DXd), a next-generation antibody-drug conjugate, combines HER2-targeted therapy with a potent cytotoxic payload, enabling specific tumor cell eradication and addressing intratumoral heterogeneity through its bystander effect. This case report details the treatment of a 69-year-old male with advanced *HER2* exon 20-mutant LUAD and widespread metastases. Following the family’s refusal of chemotherapy, T-DXd was initiated, leading to prolonged disease stabilization over 14 treatment cycles, demonstrating a progression-free survival of at least 13 months. Imaging assessments revealed a consistent reduction in the primary lung lesion size, with stable disease (SD) observed according to RECIST criteria. Despite a mixed response in brain metastases, T-DXd demonstrated a favorable safety profile without significant adverse events. While pivotal trials such as DESTINY-Lung02 and DESTINY-Lung05 have demonstrated robust efficacy and survival benefits of T-DXd as subsequent lines of therapy, our patient’s tolerance and sustained SD support the therapeutic potential of T-DXd as a first line treatment in certain real-world scenarios. In addition, this case reinforces the importance of molecular profiling in guiding personalized treatment strategies and highlights the need for further research, particularly in overcoming challenges related to central nervous system metastases. Overall, T-DXd represents a promising advancement in HER2-mutant NSCLC management, offering hope for improved outcomes in this underserved patient population.

## Introduction

Non-small cell lung cancer (NSCLC) is a leading cause of cancer-related mortality worldwide (Bray et al., 2024), and among its various subtypes, lung adenocarcinoma (LUAD) is the most prevalent. HER2 (human epidermal growth factor receptor 2) mutations represent a rare but significant oncogenic driver primarily observed in LUAD. These mutations, often located in exon 20, are present in approximately 2%–4% of NSCLC cases (Cheng et al., 2016; Harada et al., 2023). Clinically, HER2-mutant NSCLC is characterized by its aggressive behavior, including rapid disease progression, high metastatic potential and poor overall prognosis (Tan and Tan, 2022). Thus, response to conventional treatment modalities, such as platinum-based chemotherapy and immune checkpoint inhibitors, remains suboptimal in this subset of patients (Wu et al., 2019).

Although tyrosine kinase inhibitors (TKIs) targeting HER2, such as afatinib and neratinib, have shown some activity, their efficacy is often hindered by incomplete inhibition of HER2 signaling and the rapid emergence of resistance (Cooper and Gainor, 2022). Additionally, monoclonal antibody-based therapies, such as trastuzumab, have shown limited clinical benefits in HER2-mutant NSCLC when used as monotherapy (Cooper and Gainor, 2022), likely due to insufficient tumor penetration and inadequate downstream signaling blockade. To address these limitations, dual approaches combining TKIs or antibodies with chemotherapy have been explored, but these strategies often result in significant toxicity, which restricts their clinical applicability (Mazieres et al., 2016; Cooper and Gainor, 2022). Overall, the limitations of current treatment options highlight the pressing need for novel and more effective strategies to specifically target the unique molecular and structural characteristics of HER2-mutant NSCLC.

In this regard, Trastuzumab deruxtecan (T-DXd) represents a groundbreaking advancement in the treatment of HER2-mutant NSCLC (Kou et al., 2023). As an antibody-drug conjugate (ADC), T-DXd combines a monoclonal antibody targeting HER2 with a potent cytotoxic payload, enabling highly specific delivery of chemotherapy to HER2-expressing tumor cells, thereby overcoming several limitations associated with traditional HER2-directed therapies (Trail et al., 2018). A key feature of T-DXd is its high drug-to-antibody ratio (DAR), which ensures a strong cytotoxic effect, even in tumors with moderate HER2 expression levels (Nakada et al., 2019). Moreover, T-DXd introduces a “bystander effect” (Bartsch, 2020), wherein the released payload diffuses into neighboring tumor cells, irrespective of HER2 expression, and this is particularly advantageous in heterogeneous tumors, such as HER2-mutant NSCLC, where intratumoral heterogeneity and varying levels of HER2 expression often leads to treatment failure (Hinohara and Polyak, 2019). Thus, by targeting HER2-positive tumor cell populations, T-DXd provides a more comprehensive approach to tumor eradication, particularly in advanced HER2-mutant NSCLC, where conventional therapies have shown limited success.

Herein, we present the case of a patient with advanced HER2 exon 20-mutated LUAD, who despite the extensive metastases, the family refused chemotherapy due to concerns over treatment-related toxicities. Based on the latest recommendations from the Chinese Society of Clinical Oncology (CSCO) and National Comprehensive Cancer Network (NCCN) guidelines, T-DXd was proposed as a targeted therapeutic option (Li B. T. et al., 2023; Riely and Wood, 2024). His prolonged stable disease and excellent tolerance to the treatment highlight the potential of T-DXd as a first-line therapy, especially in real-world scenarios where conventional therapies may not be feasible.

## Case presentation

A 69-year-old male patient (weight: 69 kg; height: 160 cm) presented with a lung nodule detected during a routine health examination in October 2023. His medical history included grade 3 hypertension (diagnosed in 2010; highest recorded: 155/115 mmHg) that could be controlled with nifedipine sustained-release tablets and a prior radical prostatectomy for acinar adenocarcinoma of the prostate in June 2022 plus adjuvant therapy with goserelin and bicalutamide. He had no history of smoking and drinking.

### Clinical findings and diagnosis

On physical examination, the patient had a Performance Status (PS) score of 0, and no palpable lymphadenopathy was detected in the cervical, supraclavicular or axillary regions. Cardiopulmonary examination was unremarkable, with normal heart sounds and clear lung fields upon auscultation.

**FIGURE 1:**
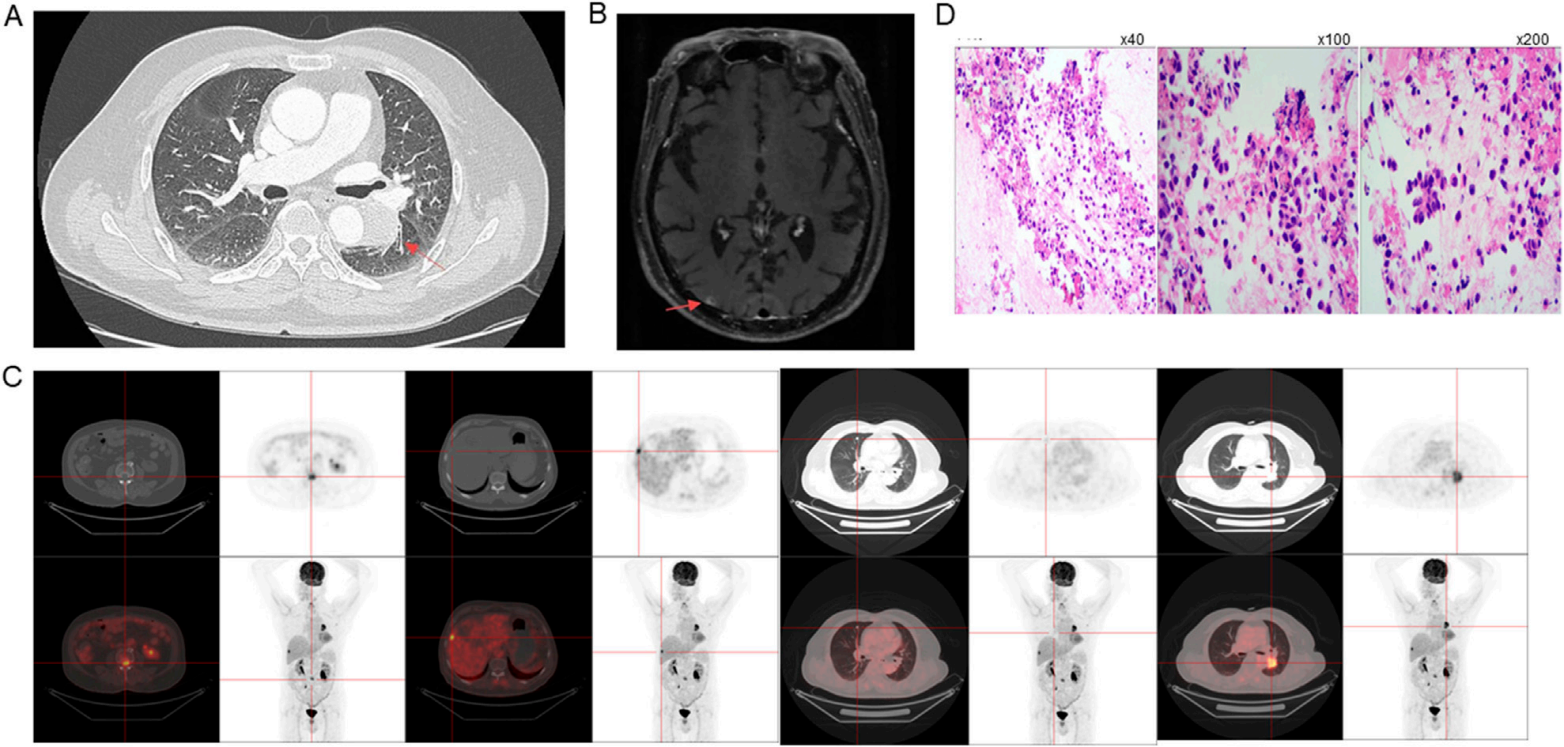
Imaging, Histopathology and Molecular Diagnostics. Initial tumor sizes at diagnosis on **(A)** CT, **(B)** MRI, and **(C)** PET-CT. **(D)** Histopathological characterization of the tumor, with immunohistochemical staining showing positivity for cytokeratin (CK) (left panel, magnification ×40), TTF-1 (middle panel, magnification ×100) and Napsin A (right panel, magnification ×200), indicative of pulmonary adenocarcinoma origin.

To assess the extent of the disease, comprehensive imaging examinations were performed. On 4 October 2023, a chest, abdominal, and pelvic computed tomography (CT) scan revealed a 29 × 35 × 39 mm soft tissue mass in the left lower lobe near the pulmonary hilum (Figure 1A). Additional findings included multiple metastatic lesions in both lungs, with the largest in the right middle lobe measuring approximately 7 × 7 mm, a bone lesion in the third lumbar vertebra (L3), appearing as a 5 × 6 mm osteolytic lesion. Subsequent magnetic resonance imaging (MRI) scan of the brain demonstrated multiple abnormal enhancing nodules in both cerebral and cerebellar hemispheres, with the largest lesion in the right cerebellar region, measuring approximately ×85 mm (Figure 1B). Additional findings included mild white matter changes in the periventricular region consistent with Fazekas grade II, indicative of age-related brain alterations, thickening of the bilateral tentorium cerebelli, with associated T2-weighted hyperintensities in the right tentorium, measuring about 9 × 3 mm. These findings suggested metastatic lesions and associated structural abnormalities. Positron emission tomography-CT (PET-CT) scan confirmed the presence of multiple metastases in the lungs, liver and bones (Figure 1C).

To obtain a definitive diagnosis, a bronchoscopy with endobronchial ultrasound-guided transbronchial needle aspiration (EBUS-TBNA) of a hilar lymph node (LN station 11) on the left side near the lower respiratory tract was performed on 12 October 2023, and histopathological examination revealed the presence of metastatic LUAD. Immunohistochemical staining demonstrated positivity for cytokeratin (CK), thyroid transcription factor-1 (TTF-1) and Napsin A, which are markers indicative of pulmonary adenocarcinoma origin (Figure 1D). The tumor cells were negative for NKX3.1, prostate-specific antigen (PSA), and P40, ruling out prostate origin and squamous differentiation. The Ki-67 proliferation index was approximately 5%, suggesting a moderate rate of tumor cell proliferation. Fluorescent amplification-refractory mutation system (ARMS)-PCR analysis of mutations in nine genes of lung cancer on 18 October 2023, detected *HER2* exon 20 insertion mutation (A775_G776insYVMA), which is known to be associated with aggressive tumor behavior, and found no *EGFR* mutations and no *ALK*, *ROS1*, or *RET* rearrangement, and others.

### Treatments

**FIGURE 2:**
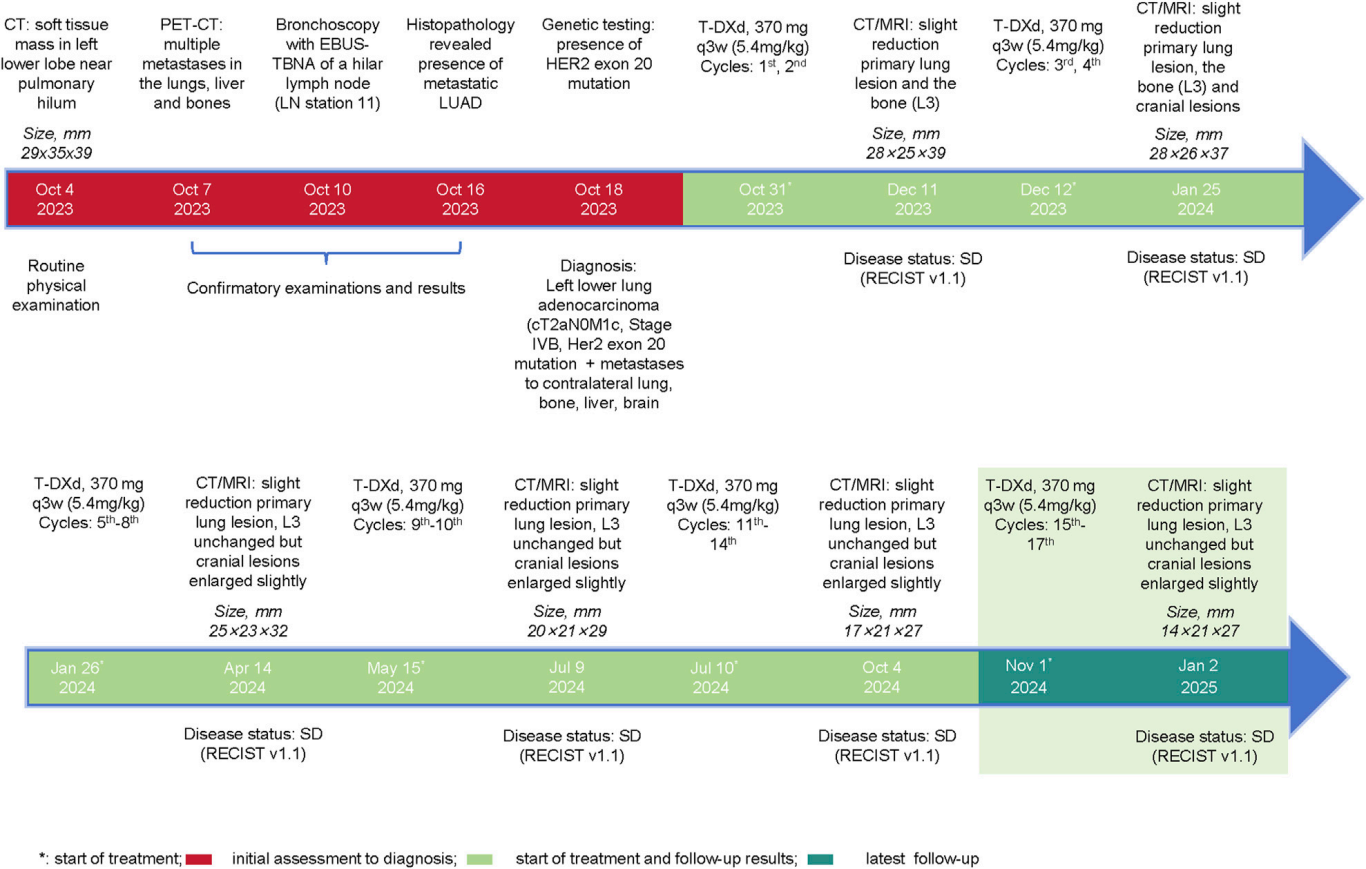
Events Timeline. Depiction of the clinical course of the patient, including initial diagnosis, imaging findings, initiation of Trastuzumab Deruxtecan (T-DXd) therapy, follow-up imaging results, and overall treatment outcomes over the 14 treatment cycles.

Given the advanced stage of the disease, classified as cT2aN0M1c (stage IVB), and considering that the patient was unaware of his diagnosis, with his family strongly opposed to chemotherapy due to concerns of treatment-related toxicities, a decision was made to administer targeted therapy, and on 31 October 2023, he was initiated on 370 mg T-DXd (5.4 mg/kg) every 3 weeks. The timeline is shown in Figure 2.

The patient’s response to therapy was closely monitored through regular imaging assessments. After two cycles of treatment (11 December 2023), CT scans of the chest, abdomen and pelvis revealed a slight reduction in the size of the primary lung lesions (largest lesion: 28 × 25 × 39 mm), slight reduction in the size of the L3 lesion, and no significant progression in the other lesions. The brain MRI showed a slight decrease in the size of the metastatic lesions, and according to both the Response Evaluation Criteria in Solid Tumors version 1.1 (RECIST v1.1) and the Response Assessment in Neuro-Oncology (RANO) criteria, the patient was evaluated to have a stable disease (SD). After the fourth cycle, on 25 January 2024, CT and MRI scan demonstrated a continued slight reduction in the size and number of the lungs (largest lesion: 28 × 26 × 37 mm), L3 and cerebral lesions, and the patient remained as SD as per the RECIST v1.1 and RANO criteria.

After eight cycles of treatment, on 14 April 2024, imaging showed that the primary lung lesions had further reduced in size to 25 × 23 × 32 mm, while the L3 lesion size remained unchanged and the brain metastases had slightly increased in both number and size (largest lesion: 9 × 3 mm). Overall, the disease status was SD.

**FIGURE 3:**
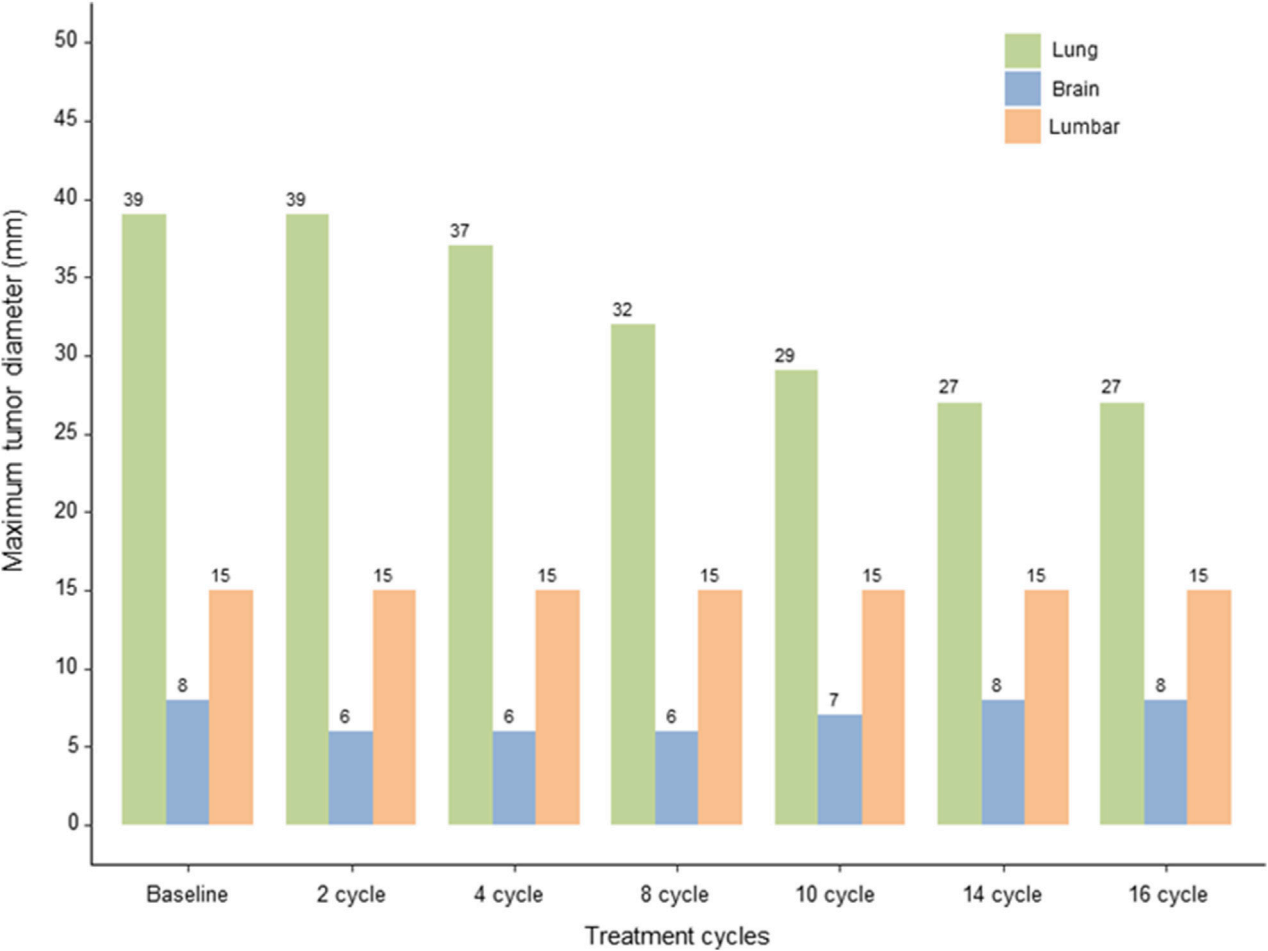
Response Evaluation. Graphical representation of the changes in the size of the primary lung lesion, lumbar and brain metastases over 14 cycles of T-DXd therapy, evaluated using the RECIST v1.1 and RANO criteria, highlighting sustained stable disease and mixed responses in brain metastases.

After the 10th cycle, on 9 July 2024, a mixed response was observed. While some lesions showed slight enlargement, others remained stable or continued to decrease in size, with the primary largest lung lesion reducing to 20 × 21 × 29 mm. Despite these variations, the disease was still evaluated as SD according to the RECIST v1.1 criteria. After completing 14 cycles of T-DXd, on 4 October 2024, the primary lung lesion was found to show a slight but continuous decrease in size to 17 × 21 × 27 mm, while the L3 lesion size remained unchanged, and the brain metastases had slightly enlarged compared to previous assessments, but the number of metastatic lesions remained unchanged. Nevertheless, the overall evaluation was considered to be progressive response. The overall changes in the lesions from baseline are shown in Figure 3.

Throughout the treatment course, the patient tolerated the therapy well, with no significant adverse events reported. Regular blood tests, including complete blood counts and biochemical profiles, remained within normal limits, indicating a favorable safety profile for the administered therapy. CT enhanced chest scan, ECG and echocardiography as well as laboratory investigations found no evidence of T-DXd related interstitial lung disease and cardiotoxicity.

## Discussion

For this 69-year-old male patient with advanced HER2 exon 20 mutated NSCLC and widespread metastases to the lungs, liver, bones and brain, T-DXd demonstrated promising efficacy and safety for more than 13 months since diagnosis. IHC staining revealed the tumor’s pulmonary origin and ruled out a prostate origin, and genetic testing identified a HER2 exon 20 mutation, which is associated with aggressive tumor behavior and poor prognosis (Friedlaender et al., 2022), while the Ki-67 proliferation index of 5% indicated moderate tumor cell proliferation, suggesting an active but not highly aggressive tumor growth rate. Together, these emphasized the significance of determining molecular characteristics of a tumor for individualized treatment, which has been particularly effective for this patient.

T-DXd, a next-generation ADC, comprises a HER2-targeting monoclonal antibody linked to the cytotoxic payload deruxtecan via a stable linker, enabling targeted delivery to HER2-expressing tumor cells. Its unique bystander effect allows the payload to impact neighboring tumor cells with lower HER2 expression, which improves efficacy even in heterogeneous tumors (Yver et al., 2020). Compared to previous generations of HER2-targeted therapies, T-DXd offers enhanced potency, improved tumor penetration and reduced off-target toxicity (Nakada et al., 2019), which might have led to the observed good therapeutic outcomes of our patient, thereby addressing one of the major challenges of traditional therapies-overcoming tumor heterogeneity.

The clinical efficacy of T-DXd in HER2-mutant NSCLC has been demonstrated in several trials such as DESTINY-Lung01 (Li et al., 2022), DESTINY-Lung02 (Goto et al., 2023), and DESTINY-Lung05 (Cheng et al., 2024). In DESTINY-Lung01 (Li et al., 2022), T-DXd achieved promising objective response rate (ORR) in patients with HER2-mutant metastatic NSCLC and superior to that typically observed with standard therapies. Similarly, DESTINY-Lung02 (Goto et al., 2023), which focused on heavily pretreated patients, reported an ORR of 50% in those patients receiving 5.4 mg/kg dose, with 1.0% complete responses and 48.0% partial responses, highlighting its effectiveness in a challenging patient population. In terms of progression-free survival (PFS), the latest data from DESTINY-Lung02 presented at the 2024 American Society of Clinical Oncology (ASCO) meeting reported a median PFS of 10 months and an overall survival (OS) of 19 months, which highlights the significant efficacy of T-DXd compared to the approximate ≤7 months of OS typically observed with conventional chemotherapy (Cooper and Gainor, 2022). Due to these compelling results, T-DXd was approved by the US Food and Drug Administration (Phillips, 2024). Following this, the DESTINY-Lung05 trial, a multi-center, open-label Phase 2 study conducted in China further reinforced the efficacy of T-DXd in HER2-mutant metastatic non-squamous NSCLC after disease progression on at least one prior line of treatment, which led to its Grade II recommendation in subsequent-line HER2-mutant NSCLC treatment in the CSCO 2024 guidelines and continued recommendation as the preferred therapy for subsequent-line treatment of HER2-mutant NSCLC in both adenocarcinoma and squamous cell carcinoma subtypes by the NCCN Guidelines (2024 V2) (Riely and Wood, 2024), thereby positioning T-DXd as a key therapeutic option for patients with limited treatment choices.

Although T-DXd has not yet received an approved indication in the first line setting for HER2 mutant NSCLC, the DESTINY-Lung04 study in first line setting is ongoing, and its data have not yet been published. However, given the promising efficacy of T-DXd in second-line and later treatments, as well as the family’s refusal of chemotherapy, we discussed with the family and decided to use T-DXd as a first-line treatment. In China, close relatives often choose not to disclose a cancer diagnosis to patients due to cultural values that emphasize protecting loved ones from emotional distress (Liu et al., 2018). Families believe that knowing the diagnosis could harm the patient’s mental and physical health; therefore, they often prioritize the patient’s perceived wellbeing over full transparency (Kao and Goh, 2013). In this present case, the patient’s family also strongly opposed chemotherapy and thus T-DXd was prescribed. He demonstrated sustained SD over 14 cycles, aligning with the promising outcomes observed in clinical trials, which provided essential insights into the potential efficacy of T-DXd in the first line treatment for HER2 mutant NSCLC.

DESTINY-Lung01 reported a median PFS of 7.1 months (95% CI, 5.5–9.8) and a median OS of 13.8 months (95% CI, 9.8–20.9) for patients with central nervous system (CNS) involvement. Moreover, exploratory pooled analyses from DESTINY-Lung01 and DESTINY-Lung02 revealed that among patients with measurable BM, T-DXd reduced BM size in approximately half of those receiving the 5.4 mg/kg dose and one-third of those receiving the 6.4 mg/kg dose, with complete resolution observed in three cases (Li Yingge et al., 2023). Though T-DXd has promising intracranial antitumor activities, the molecular mechanism underlying its CNS activity remains elusive. Trastuzumab administered intravenously reaches the cerebrospinal fluid in the CNS at only 1%–10% of its serum concentration (Stemmler et al., 2007). The variable response in brain metastases, which might be due to its limited ability to penetrate the blood brain barrier or tumor heterogeneity, suggests the need for a combination approach such as intracranial radiotherapy or the concurrent use of CNS penetrant small molecule inhibitor. However, data on the impact of non-selective HER2 TKIs like afatinib, dacomitinib, and pyrotinib on the central nervous system (CNS) are lacking.

Despite that safety profiles were similar between patients with and without BM at baseline, grade ≥3 and serious adverse events were more frequent in those with BM. Comparatively, the brain metastases of our patient demonstrated mixed responses, which may suggest limitations in T-DXd to fully penetrate the blood-brain barrier or tumor heterogeneity, raising considerations for further improvement of the drug’s efficacy through combination with other drugs or radiotherapy for such cases. Despite this, our patient experienced no significant adverse events, demonstrating T-DXd’s tolerability even after prolonged treatment, particularly for those who may not be candidates for more aggressive chemotherapy regimens.

In conclusion, this case suggests that T-DXd may be a feasible first-line option in select patients, but further clinical evidence is needed. Given the promising efficacy of T-DXd, there is a clear need for further research, particularly in exploring combination strategies and addressing challenges such as CNS metastasis control, as observed in our patient due to the mixed responses in brain lesions. Future trials could investigate ways to improve CNS penetration and explore novel combination regimens to further improve treatment responses and quality of life.

## Data Availability

The original contributions presented in the study are included in the article/supplementary material, further inquiries can be directed to the corresponding author.

## Abbreviations

ADC, Antibody-Drug Conjugate; CSCO, Chinese Society of Clinical Oncology; CT, Computed Tomography; CNS, Central Nervous System; EBUS-TBNA, Endobronchial Ultrasound-Guided Transbronchial Needle Aspiration; HER2, Human Epidermal Growth Factor Receptor 2; IHC, Immunohistochemistry; LUAD, Lung Adenocarcinoma; LN, Lymph Node; MRI, Magnetic Resonance Imaging; NSCLC, Non-Small Cell Lung Cancer; NCCN, National Comprehensive Cancer Network; ORR, Objective Response Rate; OS, Overall Survival; PS, Performance Status; PET-CT, Positron Emission Tomography-Computed Tomography; PSA, Prostate-Specific Antigen; PFS, Progression-Free Survival; RECIST, Response Evaluation Criteria in Solid Tumors; T-DXd, Trastuzumab Deruxtecan; TKI, Tyrosine Kinase Inhibitor.

## References

[B1] Bartsch R. (2020). Trastuzumab-deruxtecan: an investigational agent for the treatment of HER2-positive breast cancer. Expert Opin. Investig. Drugs 29 (9), 901–910. 10.1080/13543784.2020.1792443 32701032

[B2] Bray F. Laversanne M. Sung H. Ferlay J. Siegel R. L. Soerjomataram I. (2024). Global cancer statistics 2022: GLOBOCAN estimates of incidence and mortality worldwide for 36 cancers in 185 countries. CA Cancer J. Clin. 74 (3), 229–263. 10.3322/caac.21834 38572751

[B3] Cheng H. Liu P. Ohlson C. Xu E. Symonds L. Isabella A. (2016). PIK3CA(H1047R)- and Her2-initiated mammary tumors escape PI3K dependency by compensatory activation of MEK-ERK signaling. Oncogene 35 (23), 2961–2970. 10.1038/onc.2015.377 26640141 PMC4896860

[B4] Cheng Y. Wu L. Fang Y. Fan Y. Li X. Zhang M. (2024). Abstract CT248: trastuzumab deruxtecan (T-DXd) in Chinese patients (pts) with previously treated HER2 mutant non-small cell lung cancer (NSCLC): primary analysis from the Phase 2 DESTINY-Lung05 (DL-05) trial. Cancer Res. 84 (7_Suppl. ment), CT248. 10.1158/1538-7445.am2024-ct248

[B5] Chinese Society of Clinical Oncology (2024). Guidelines of Chinese society of clinical Oncology (CSCO): non-small cell lung cancer 2024. 1st ed. Beijing: People's Medical Publishing House.

[B6] Cooper A. J. Gainor J. F. (2022). Human epidermal growth factor receptor 2-mutant non-small-cell lung cancer: continued progress but challenges remain. J. Clin. Oncol. 40 (7), 693–697. 10.1200/JCO.21.02550 35072488

[B7] Friedlaender A. Subbiah V. Russo A. Banna G. L. Malapelle U. Rolfo C. (2022). EGFR and HER2 exon 20 insertions in solid tumours: from biology to treatment. Nat. Rev. Clin. Oncol. 19 (1), 51–69. 10.1038/s41571-021-00558-1 34561632

[B8] Goto K. Goto Y. Kubo T. Ninomiya K. Kim S. W. Planchard D. (2023). Trastuzumab deruxtecan in patients with HER2-mutant metastatic non-small-cell lung cancer: primary results from the randomized, Phase II DESTINY-lung02 trial. J. Clin. Oncol. 41 (31), 4852–4863. 10.1200/JCO.23.01361 37694347 PMC10617843

[B9] Harada G. Yang S. R. Cocco E. Drilon A. (2023). Rare molecular subtypes of lung cancer. Nat. Rev. Clin. Oncol. 20 (4), 229–249. 10.1038/s41571-023-00733-6 36806787 PMC10413877

[B10] Hinohara K. Polyak K. (2019). Intratumoral heterogeneity: more than just mutations. Trends Cell Biol. 29 (7), 569–579. 10.1016/j.tcb.2019.03.003 30987806 PMC6579620

[B11] Kao Y. H. Goh C. R. (2013). The practice of nondisclosure of advanced cancer diagnosis in Singapore: a continuing challenge. Singap. Med. J. 54 (5), 255–258. 10.11622/smedj.2013103 23716149

[B12] Kou L. Chen X. Xie X. Wen Q. Li J. Li Y. (2023). The efficacy and safety of trastuzumab deruxtecan (T-DXd) in HER2-expressing solid tumours: a single-arm meta-analysis. Jpn. J. Clin. Oncol. 53 (8), 722–729. 10.1093/jjco/hyad036 37114934

[B13] Li B. T. Goto K. Smit E. F. De Langen J. Goto Y. Ninomiya K. (2023a). 1321MO Trastuzumab deruxtecan (T-DXd) in patients (pts) with HER2 (ERBB2)-mutant (HER2m) metastatic non–small cell lung cancer (NSCLC) with and without brain metastases (BMs): pooled analyses from DESTINY-Lung01 and DESTINY-Lung02. Ann. Oncol. 34, S762–S763. 10.1016/j.annonc.2023.09.2354

[B14] Li B. T. Smit E. F. Goto Y. Nakagawa K. Udagawa H. Mazieres J. (2022). Trastuzumab deruxtecan in HER2-mutant non-small-cell lung cancer. N. Engl. J. Med. 386 (3), 241–251. 10.1056/NEJMoa2112431 34534430 PMC9066448

[B15] Li Y. Yi D. Shuyang Y. Yingmei W. Qibin S. Yi Y. (2023b). Diagnostic and therapeutic strategy updates of rare oncogenic mutations in ChineseSociety of clinical Oncology guidelines on diagnosis and treatment of non-small CellLung cancer. Cancer Res. Prev. Treat. 50 (12), 1232–1236. 2023 Edition).

[B16] Liu Y. Yang J. Huo D. Fan H. Gao Y. (2018). Disclosure of cancer diagnosis in China: the incidence, patients' situation, and different preferences between patients and their family members and related influence factors. Cancer Manag. Res. 10, 2173–2181. 10.2147/CMAR.S166437 30087577 PMC6061405

[B17] Mazieres J. Barlesi F. Filleron T. Besse B. Monnet I. Beau-Faller M. (2016). Lung cancer patients with HER2 mutations treated with chemotherapy and HER2-targeted drugs: results from the European EUHER2 cohort. Ann. Oncol. 27 (2), 281–286. 10.1093/annonc/mdv573 26598547

[B18] Nakada T. Sugihara K. Jikoh T. Abe Y. Agatsuma T. (2019). The latest research and development into the antibody-drug conjugate, [fam-] trastuzumab deruxtecan (DS-8201a), for HER2 cancer therapy. Chem. Pharm. Bull. (Tokyo) 67 (3), 173–185. 10.1248/cpb.c18-00744 30827997

[B19] Phillips C. (2024). FDA approves trastuzumab deruxtecan for any HER2-positive solid cancer.

[B20] Riely G. J. Wood D. E. (2024). “Non-small cell lung cancer,” in *NCCN clinical practice Guidelines in Oncology (NCCN Guidelines®)* (11.2024).

[B21] Stemmler H. J. Schmitt M. Willems A. Bernhard H. Harbeck N. Heinemann V. (2007). Ratio of trastuzumab levels in serum and cerebrospinal fluid is altered in HER2-positive breast cancer patients with brain metastases and impairment of blood-brain barrier. Anticancer Drugs 18 (1), 23–28. 10.1097/01.cad.0000236313.50833.ee 17159499

[B22] Tan A. C. Tan D. S. W. (2022). Targeted therapies for lung cancer patients with oncogenic driver molecular alterations. J. Clin. Oncol. 40 (6), 611–625. 10.1200/JCO.21.01626 34985916

[B23] Trail P. A. Dubowchik G. M. Lowinger T. B. (2018). Antibody drug conjugates for treatment of breast cancer: novel targets and diverse approaches in ADC design. Pharmacol. Ther. 181, 126–142. 10.1016/j.pharmthera.2017.07.013 28757155

[B24] Wu Y. L. Planchard D. Lu S. Sun H. Yamamoto N. Kim D. W. (2019). Pan-Asian adapted Clinical Practice Guidelines for the management of patients with metastatic non-small-cell lung cancer: a CSCO-ESMO initiative endorsed by JSMO, KSMO, MOS, SSO and TOS. Ann. Oncol. 30 (2), 171–210. 10.1093/annonc/mdy554 30596843

[B25] Yver A. Agatsuma T. Soria J. C. (2020). The art of innovation: clinical development of trastuzumab deruxtecan and redefining how antibody-drug conjugates target HER2-positive cancers. Ann. Oncol. 31 (3), 430–434. 10.1016/j.annonc.2019.11.019 32067685

